# The cytochrome *bd*-type quinol oxidase is important for survival of *Mycobacterium smegmatis* under peroxide and antibiotic-induced stress

**DOI:** 10.1038/srep10333

**Published:** 2015-05-27

**Authors:** Ping Lu, Marieke H. Heineke, Anil Koul, Koen Andries, Gregory M. Cook, Holger Lill, Rob van Spanning, Dirk Bald

**Affiliations:** 1 Department of Molecular Cell Biology, Amsterdam Institute for Molecules, Medicines and Systems, Faculty of Earth- and Life Sciences, VU University Amsterdam, De Boelelaan 1085, 1081 HV Amsterdam, The Netherlands; 2 Infectious diseases and vaccines therapeutic area, Janssen Research & Development, Johnson & Johnson Pharmaceuticals, Turnhoutseweg 30, 2340-Beerse, Belgium; 3 Department of Microbiology and Immunology, Otago School of Medical Sciences, University of Otago, Dunedin 9054, New Zealand

## Abstract

Targeting respiration and ATP synthesis has received strong interest as a new strategy for combatting drug-resistant *Mycobacterium tuberculosis*. Mycobacteria employ a respiratory chain terminating with two branches. One of the branches includes a cytochrome *bc*_1_ complex and an *aa*_3_-type cytochrome c oxidase while the other branch terminates with a cytochrome *bd*-type quinol oxidase. In this communication we show that genetic inactivation of cytochrome *bd*, but not of cytochrome *bc*_1_, enhances the susceptibility of *Mycobacterium smegmatis* to hydrogen peroxide and antibiotic-induced stress. The type-II NADH dehydrogenase effector clofazimine and the ATP synthase inhibitor bedaquiline were bacteriostatic against wild-type *M. smegmatis*, but strongly bactericidal against a cytochrome *bd* mutant. We also demonstrated that the quinone-analog aurachin D inhibited mycobacterial cytochrome *bd* at sub-micromolar concentrations. Our results identify cytochrome *bd* as a key survival factor in *M. smegmatis* during antibiotic stress. Targeting the cytochrome *bd* respiratory branch therefore appears to be a promising strategy that may enhance the bactericidal activity of existing tuberculosis drugs.

*Mycobacterium tuberculosis* is the causative agent of tuberculosis disease (TB). In 2013 there were 1.5 million TB-related deaths worldwide and 9 million people were newly infected with TB^1^. Despite the introduction of efficient antibiotics in the 1950 s, TB treatment remains challenging, largely due to the emergence of drug-resistant strains^2^,^3^. Additionally, its metabolic flexibility allows the pathogen to exist in different states, ranging from actively replicating to dormant persisting^4^,^5^. The dormant population is difficult to eradicate and has the potential to cause active tuberculosis after resuscitation, which is especially threatening for immune-compromised patients suffering from HIV^6^. Therefore, drugs with novel mechanisms of action are urgently needed to adequately kill the heterogeneous population of bacteria and to counter multi-drug resistant (MDR) and extensively-drug resistant (XDR) tuberculosis strains. Since basal energy requirements and redox balance are essential for both replicating and persisting bacteria, components of the oxidative phosphorylation pathway are regarded as promising drug targets^7^,^8^,^9^,^10^,^11^.

The respiratory chain enzyme complexes that are part of the oxidative phosphorylation pathway establish a proton motive force across the bacterial cytoplasmic membrane and ATP synthase utilizes the energy of the proton motive force for synthesis of ATP. Mycobacterial ATP synthase has been validated as target of bedaquiline (BDQ), the lead compound of the diarylquinoline class of drugs, which selectively inhibits this enzyme in a variety of mycobacterial strains^12^,^13^,^14^,^15^,^16^. BDQ has received accelerated approval by the US Food & Drug Administration (FDA) and the European Medicines Agency (EMA) for treatment of MDR-TB^17^,^18^. Moreover, components of the respiratory chain such as the type-II NADH dehydrogenase (NDH-2) and the cytochrome *bc*_1_ complex are targeted by small-molecule compounds that are currently in clinical development^19^,^20^,^21^,^22^,^23^,^24^. Mycobacteria have a branched electron transport chain. Electrons from the menaquinone pool can be passed on either to the cytochrome *bc*_1_ complex, which forms a supercomplex with the cytochrome *aa*_3_ oxidase, or alternatively to the cytochrome *bd*-type quinol oxidase^9^,^10^,^20^ (Fig. 1). Both branches transfer the electrons onto molecular oxygen, yielding H_2_O, but they differ in the efficiency of energy conservation. The cytochrome *bc*_1_/*aa*_3_ branch establishes a higher proton motive force as compared with the cytochrome *bd* branch and consequently is energetically more efficient. Therefore, this respiratory branch is mainly utilized during aerobic, replicating conditions^25^,^26^.

Genetic knock-out of the cytochrome *bc*_1_ complex in *M. smegmatis* substantially decreased the growth rate of the bacteria under aerobic growth conditions, while knock-out of cytochrome *bd* did not^25^,^27^. The cytochrome *bc*_1_ complex has also been validated as target of the imidazopyridine class of drugs^22^,^24^, whereas no antibacterials targeting cytochrome *bd* have been reported yet. These findings point towards cytochrome *bc*_1_/*aa*_3_ as the more promising drug target of the two respiratory chain branches in mycobacteria. However, the proteins of the cytochrome *bc*_1_/*aa*_3_ branch are down regulated during hypoxia and chronic infection in a mouse model, while these conditions induced the expression of cytochrome *bd*, suggesting an important role for this enzyme in respiration during hypoxia^26^. Additionally, cytochrome *bd* was induced when the cytochrome *bc*_1_ complex was impaired due to deletion mutations^25^, upon inhibition by small-molecule drugs^28^ or when cytochrome c maturation was disturbed^29^, suggesting that the cytochrome *bd* branch may (partially) be able to compensate for lack of function of the cytochrome *bc*_1_/*aa*_3_ branch of the respiratory chain^25^,^28^,^29^.

Cytochrome *bd* can also play a role in protection against different types of stress^30^,^31^,^32^. In *Escherichia coli*, exposure to exogenous hydrogen peroxide and nitric oxide induced expression of cytochrome *bd* and strains lacking cytochrome *bd* were found hyper-sensitive to peroxide and nitrosative stress^33^,^34^,^35^,^36^ as well as to low iron concentrations^37^. In *M. tuberculosis*, cytochrome *bd* expression in the mouse lung is upregulated during chronic infection^26^. During an inflammatory reaction, macrophages in the host can produce reactive oxygen species (ROS) to kill engulfed bacteria. Overexpression of cytochrome *bd* in *M. tuberculosis* is associated with increased peroxide resistance^29^. Upregulation of cytochrome *bd* may represent a protection mechanism to survive the host’s immune response. These data point towards cytochrome *bd* as an important contributor to stress resistance in (myco-) bacteria.

In this study, the role of the two mycobacterial respiratory chain branches in response to stress elicited by peroxides and antimicrobials was investigated. For this aim we challenged strains of *M. smegmatis* lacking cytochrome *bd* or the cytochrome *bc*_1_ complex *in vitro* with these stress factors to elucidate the importance of each respiratory chain branch in protection against them.

## Results

### Bioenergetic parameters of *Mycobacterium smegmatis* strains with inactivated respiratory chain branches

**Figure 1:**
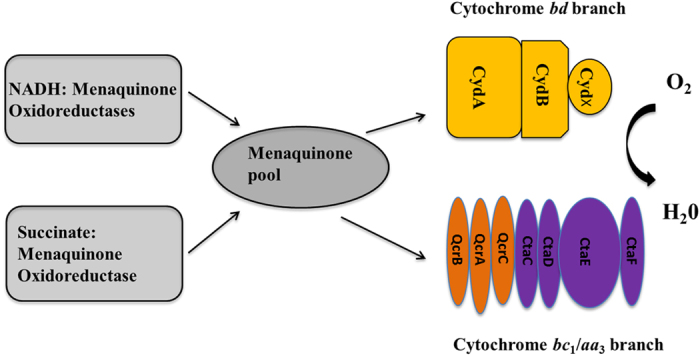
The branched respiratory chain in mycobacteria. Cyd: cytochrome *bd*-type quinol oxidase subunits, Qcr: cytochrome *bc*_1_ complex subunits, Cta: subunits of *aa*_3_-type cytochrome *c* oxidase. Note that *M. smegmatis* does not have a soluble cytochrome *c*. Instead QcrC is a di-heme cytochrome *c*, which transfers electrons between the cytochrome *bc*_1_ complex and the *aa*_3_-type cytochrome *c* oxidase.

**Figure 2:**
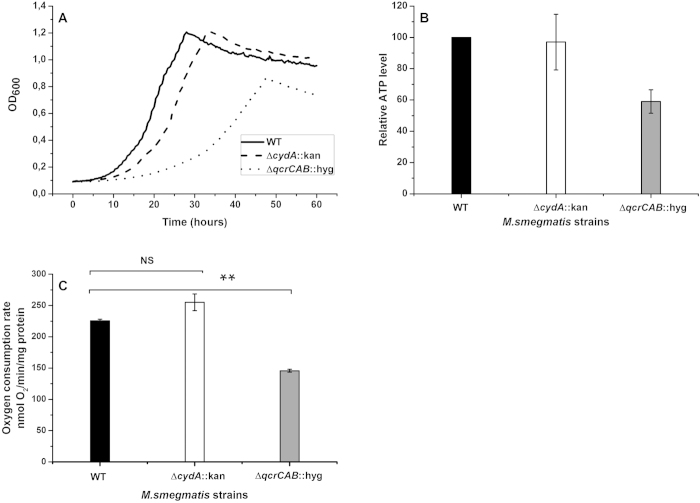
Bioenergetic properties of *M. smegmatis* strains lacking the cytochrome *bd* or the cytochrome *bc*_1_ complex. (**A**) Wild-type (WT) and mutant strains with knocked-out cytochrome *bc*_1_ complex (Δ*qcrCAB* :: hyg) or cytochrome *bd* (Δ*cydA* :: kan) were grown overnight, sub-cultured in fresh medium and incubated at 37 °C for 60 h. The optical density at 600 nm was measured in 20 min intervals. Data are representative of two independent experiments, each done in triplicate. (**B**) Cellular ATP levels in WT and mutant *M. smegmatis* as determined by the Luciferase method. (**C**) Oxygen consumption rates of inverted membrane vesicles from wild-type and mutant *M. smegmatis* strains using NADH as substrate. Data represent average plus standard error of the mean (SEM) for one experiment done threefold. One-way ANOVA was used for statistical analysis, NS: not significant (P value > 0.05), ** represent P value < 0.01.

The role of two respiratory chain branches in mycobacteria was investigated using mutant strains impaired in one of the two branches. These strains maintain either only the cytochrome *bd* branch (strain Δ*qcrCAB* :: hyg) or the cytochrome *bc*_1_/*aa*_3_ branch (strain Δ*cydA* :: kan) (Fig. 1). The growth rate of the Δ*cydA* :: kan strain was comparable to that of the wild-type, whereas growth of the Δ*qcrCAB* :: hyg strain was substantially lower (Fig. 2A), confirming previous data^25^,^27^. We then extended the earlier reported microbiological characterization of the mutant strains and determined central bioenergetic parameters for the two mutants. Cellular ATP levels were unaltered in the Δ*cydA* :: kan mutant as compared with the wild-type, but were decreased by ~40% in the Δ*qcrCAB* :: hyg strain (Fig. 2B). Similarly, oxygen consumption rates in inverted membrane vesicles isolated form aerobically grown cells were almost unchanged in the Δ*cydA* :: kan mutant versus wild-type, but lower in Δ*qcrCAB* :: hyg (Fig. 2C). These results reflect the higher respiratory efficiency of the cytochrome *bc*_1_/*aa*_3_ branch. Based on growth rate and bioenergetic characterization the cytochrome *bc*_1_/*aa*_3_ branch can be regarded as the more promising target pathway of the two branches.

### Sensitivity for hydrogen peroxide stress

**Figure 3:**
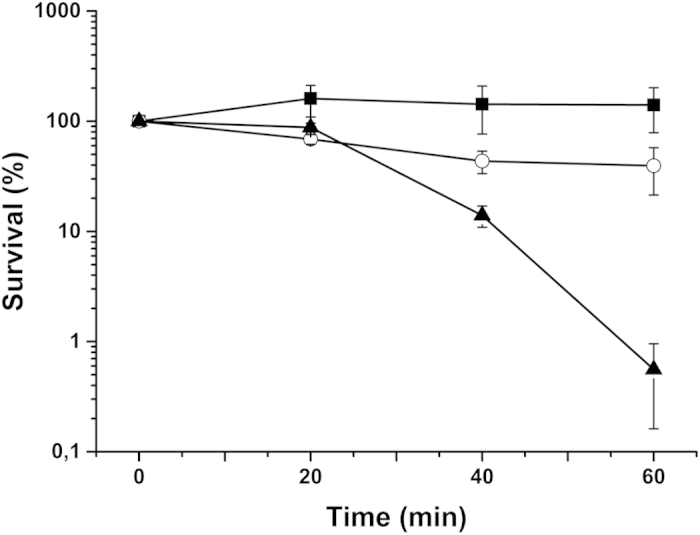
Sensitivity for hydrogen peroxide of *Mycobacterium smegmatis* respiratory chain mutants. The effect of hydrogen peroxide (20 mM) on the survival of exponentially growing *M. smegmatis* is shown: wild-type (filled squares), Δ*cydA* :: kan (filled triangles), and Δ*qcrCAB* :: hyg (open circle). Results represent means of two independent experiments with standard error of the mean (SEM).

Next, we investigated the importance of the two respiratory chain branches in response to peroxide stress. Exponentially growing *M. smegmatis* cells were exposed to hydrogen peroxide (20 mM, final conc.) for various time intervals and colony-forming units were enumerated. Incubation with hydrogen peroxide had a bacteriostatic effect on wild-type *M. smegmatis* and for the Δ*qcrCAB* :: hyg mutant a minor decrease in viability was found (Fig. 3). For the Δ*cydA* :: kan mutant, a 99% decline in cell viability was observed after 60 min exposure (Fig. 3). These results suggest that cytochrome *bd* plays a protective role during oxidative stress in *M. smegmatis*, whereas the cytochrome *bc*_1_ complex is of minor importance for survival under these conditions.

### Sensitivity for the NDH-2 effector clofazimine

**Figure 4:**
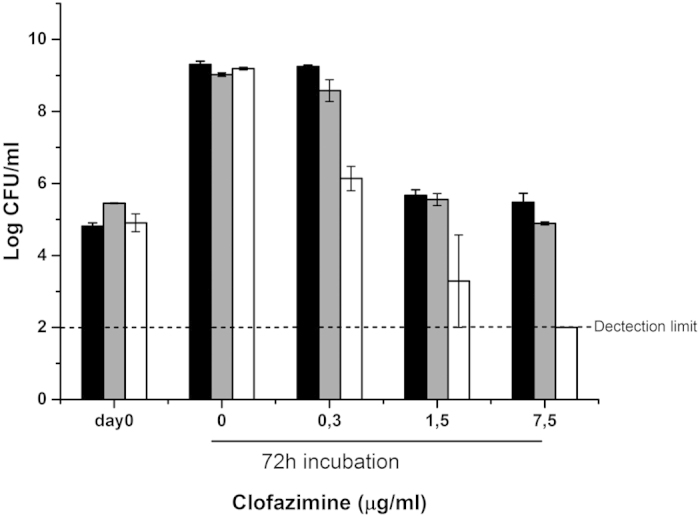
Impact of *Mycobacterium smegmatis* respiratory chain mutations on the susceptibility for clofazimine. Strains of *M. smegmatis* were treated with the indicated amounts of clofazimine for 72 hours and CFU/mL were counted on agar plates after three (wild-type, Δ*cydA* :: kan) or four days (Δ*qcrCAB* :: hyg) of incubation. Black bars, wild-type; grey bars: Δ*qcrCAB* :: hyg; white bars: Δ*cydA* :: kan. Error bars represent means of at least two independent experiments with standard error of the mean (SEM).

We hypothesized that mycobacteria with impaired respiratory chain branches may also be more sensitive to antimicrobials that cause production of reactive oxygen species. Clofazimine (CFZ) is a front-line anti-leprosy drug that presently is repurposed for usage against tuberculosis. CFZ is an electron carrier that interferes with the type II NADH dehydrogenase (NDH-2) in mycobacteria^19^. As such, it can transfer electrons from NDH-2 directly to oxygen, thereby producing ROS^19^. First, we confirmed that CFZ caused time-dependent development of ROS by inverted membrane vesicles from the *M. smegmatis* wild-type strain used in our laboratory (Supplementary Figure S1). Subsequently we investigated if either cytochrome *bd* or the cytochrome *bc*_1_ complex can protect *M. smegmatis* against CFZ. For this purpose the bacteria were incubated for 72 hours in liquid culture with varying concentrations of the drug. CFZ was bacteriostatic against the wild-type strain, even at the highest concentration investigated (25x MIC, 7.5 μg/mL) (Fig. 4). The Δ*qcrCAB* :: hyg mutant showed marginally higher sensitivity for CFZ as compared with the wild-type (Fig. 4). However, the viability of the Δ*cydA* :: kan mutant was strongly reduced in response to CFZ challenge. CFZ at concentrations >0.3 μg/mL was bacteriostatic for the Δ*cydA* :: kan mutant and concentrations >1.5 μg/mL were bactericidal. With 7.5 μg/mL CFZ the limit of detection was reached after 72 hours of exposure (Fig. 4). These results indicate that cytochrome *bd*, but not the cytochrome *bc*_1_ complex, can protect the bacteria against the bactericidal effect of clofazimine. We hypothesized that the increased sensitivity of the Δ*cydA* :: kan strain was due to ROS production by CFZ. To test this hypothesis we investigated the effect of chlorpromazine (CPZ), a phenothiazine-class drug that inhibits type-II NADH dehydrogenase^20^,^23^, but does not produce ROS^19^, on wild-type and the Δ*cydA* :: kan mutant. As expected, CPZ did not discriminate between wild-type *M. smegmatis* and the Δ*cydA* :: kan mutant (Supplementary Figure S1).

### Sensitivity for the ATP synthase inhibitor bedaquiline

The results described above demonstrate that genetic inactivation of cytochrome *bd*, but not of the cytochrome *bc*_1_ complex, converts the bacteriostatic effect of hydrogen peroxide and of clofazimine into a bactericidal effect. Next, we expanded our experiments to the ATP synthase inhibitor bedaquiline (BDQ). Whereas BDQ is bactericidal against *M. tuberculosis*, it is bacteriostatic against *M. smegmatis*^12^. A transcriptional and proteomic analysis recently revealed that treatment of *M. tuberculosis* with BDQ triggers strong upregulation of cytochrome *bd*^38^ and deletion of cytochrome *bd* in *M. tuberculosis* enhanced the bactericidal activity of BDQ^39^. We therefore investigated if genetic inactivation of one of the respiratory chain branches would convert the bacteriostatic activity of BDQ on *M. smegmatis* into bactericidal activity.

**Figure 5:**
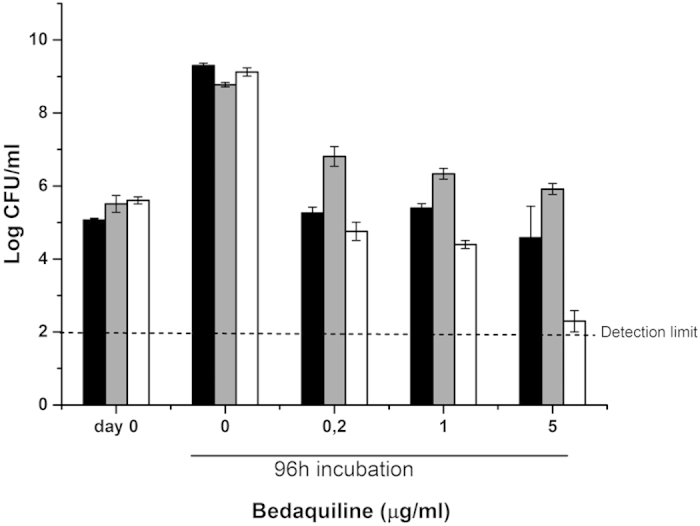
Impact of *Mycobacterium smegmatis* respiratory chain mutations on the susceptibility for bedaquiline. Strains of *M. smegmatis* were treated with indicated amounts of bedaquiline for 96 hours and CFU/mL were counted on agar plates after three (wild-type, Δ*cydA* :: kan) or four days (Δ*qcrCAB* :: hyg) of incubation at 37 °C. Black bars: wild-type; grey bars: Δ*qcrCAB* :: hyg; white bars: Δ*cydA* :: kan. Results represent the means of two independent experiments with standard error of the mean (SEM).

BDQ was bacteriostatic against wild-type *M. smegmatis*, even at the highest concentration used (300x MIC, 5 μg/mL) (Fig. 5). The Δ*qcrCAB* :: hyg strain was less sensitive to BDQ as compared with the wild-type strain (Fig. 5). However, in case of the Δ*cydA* :: kan mutant, challenge with BDQ (1 μg/mL) led to a ~1 log_10_ reduction in colony forming units and 5 μg/mL BDQ caused ~3 log_10_ kill, approaching the limit of detection after 3 days of treatment (Fig. 5). Cytochrome *bd* thus protects *M. smegmatis* against killing by bedaquiline, whereas the cytochrome *bc*_1_/*aa*_3_ branch does not. We attempted to link the protective function of cytochrome *bd* to production of ROS in the presence of BDQ, however, inverted membrane vesicles from *M. smegmatis* did not show increased ROS formation after treatment with BDQ (Supplementary Figure S1).

The results obtained for CFZ and BDQ demonstrate that inactivation of the cytochrome *bd* branch, but not of the cytochrome *bc*_1_/*aa*_3_ branch, can convert bacteriostatic activity of an antibacterial drug into bactericidal activity. Our findings identify cytochrome *bd* as an important survival factor in mycobacterial metabolism.

### Inactivation of mycobacterial cytochrome *bd* by a small-molecule inhibitor

**Figure 6:**
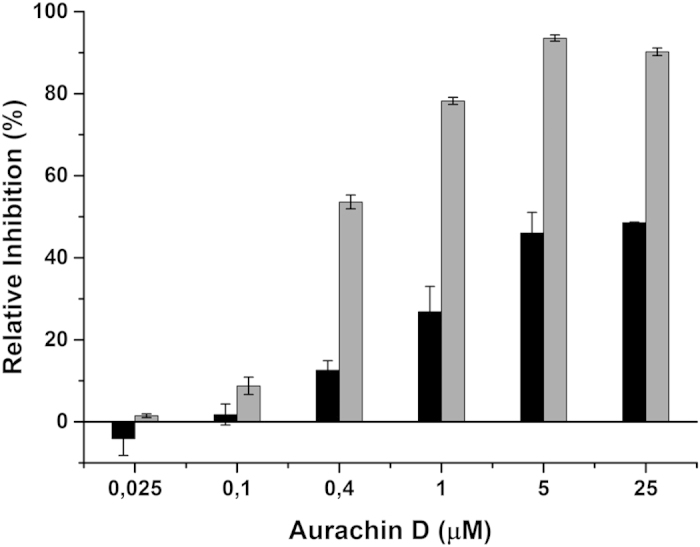
Aurachin D inhibits cytochrome *bd* activity of *Mycobacterium smegmatis* membrane vesicles. Oxygen consumption activity of inverted membrane vesicles from *M. smegmatis* was measured with a Clark-type electrode. The reaction was started by addition of NADH (250 μM final conc.) as electron donor and recorded for 90 s. Black bars: wild-type; gray bars: Δ*qcrCAB* :: hyg. Results represent the means of two independent experiments with standard error of the mean (SEM).

Genetic inactivation of cytochrome *bd* can considerably increase the potency of two prominent antibacterial drugs, CFZ and BDQ. Based on these findings we tested if small-molecule inhibitors can block the activity of cytochrome *bd* in *M. smegmatis*. The aurachin class of quinone analogs has been reported as inhibitors of a variety of quinone-modifying enzyme^40^,^41^,^42^. Within this class, aurachin D was previously shown to preferentially inhibit *E. coli* cytochrome *bd* as compared with other quinone-modifying enzymes^42^. We investigated the effect of aurachin D on the oxygen consumption activity of inverted membrane vesicles from *M. smegmatis*. Aurachin D inhibited oxygen consumption in a dose-dependent manner with 50% maximal inhibition for wild-type strain (Fig. 6). Interestingly, this inhibitory effect was clearly stronger in membrane vesicles of the Δ*qcrCAB* :: hyg strain, where ~90% maximal inhibition was reached (IC_50_ ~400 nM) (Fig. 6). This suggests that the main target in mycobacterial oxidative phosphorylation was cytochrome *bd*.

Subsequently, we evaluated the effect of aurachin D on mycobacterial growth. We found that for all three strains tested (wild-type, Δ*cydA* :: kan, Δ*qcrCAB* :: hyg) the minimal inhibitory concentrations (MICs) were >85 μM (data not shown). This result suggests that the inhibitor is not capable of effectively crossing the mycobacterial cell envelope.

## Discussion

Previously it has been reported that genetic inactivation of cytochrome *bd* considerably decreased virulence or survival in the host of a variety of pathogenic bacterial strains. In *Shigella flexneri, Brucella abortus* and *Salmonella enterica Serovar Typhymurium*, the causative agents of bacterial dysentery, brucellosis and typhoid fever, inactivation of cytochrome *bd* considerably impaired intracellular survival and virulence^43^,^44^,^45^. In *Klebsiella pneumonia* cytochrome *bd* was found crucial for free energy transduction under microaerobic conditions and for protection of anaerobic processes such as nitrogen fixation^46^. In case of group B streptococci, inactivation of cytochrome *bd* led to decreased growth in human blood^47^. Cytochrome *bd* may also allow strictly anaerobic bacteria such as *Bacteriodes fragilis* to survive under nanomolar oxygen concentrations, potentially facilitating survival of opportunistic pathogens in the host^48^.

In this study, we evaluated the function of the two mycobacterial respiratory chain branches in response to stress. The cytochrome *bc*_1_ complex is a validated drug target in *M. tuberculosis*^22^,^24^, however, upregulation of cytochrome *bd* may partially compensate for inhibition of cytochrome *bc*_1_ function^25^,^28^,^29^. Therefore, it has been postulated that simultaneously targeting both respiratory chain branches with inhibitors might be required to effectively disrupt mycobacterial respiration^29^. Whereas the cytochrome *bd* branch may in part be able to compensate for inactivation of the cytochrome *bc*_1_ complex, our results indicate that the cytochrome *bc*_1_/*aa*_3_ branch is not able to compensate for loss of cytochrome *bd* functionality. Inactivation of cytochrome *bd*, although not directly leading to a phenotype, exerts a strong impact on bacterial viability in the presence of antibiotic stress. This highlights the importance of the cytochrome *bd* branch as a survival factor in *M. smegmatis* and suggests that targeting this terminal oxidase may be a successful strategy for weakening the mycobacterial stress response.

The hypersensitivity of the *cydAB* mutants to exogenous hydrogen peroxide is not due to impaired growth of the mutant strain, since growth rate and ATP levels are similar to the wild-type. Giuffre, Borisov and colleagues suggested two molecular mechanisms for peroxide protection by cytochrome *bd* in *E.coli*^32^. First, cytochrome *bd* as oxygen scavenger may decrease the intracellular oxygen tension, thereby preventing the formation of reactive oxygen species. Second, cytochrome *bd* displays catalase activity^32^,^34^ and might thus directly metabolize peroxides. Both mechanisms may contribute to the protective role of cytochrome *bd* against hydrogen peroxide stress in *M. smegmatis* and their respective importance in mycobacteria needs to be further elucidated.

Our experiments revealed that cytochrome *bd* plays an important role in protection against two prominent anti-tuberculosis drugs, both targeting oxidative phosphorylation. Protection against clofazimine, a ROS-producing drug, is most likely due to the ability of cytochrome *bd* to metabolize and/or prevent formation of peroxides. Our data do not allow for pinpointing the mechanism of protection against BDQ. Inhibition of ATP synthase may well result in reduction of the electron flow through the respiratory system. As a result, the reduction state of the respiratory complexes increases which in turn leads to increased production of ROS. Higher cellular NADH/NAD^+^ ratios and enhanced expression of bacterioferritin, indicating BDQ-induced backpressure and ROS formation, have previously been reported for *M. tuberculosis* treated with BDQ^38^. However, it is possible that the levels of ROS produced by BDQ are not high enough for detection in case the membrane vesicles used in our study are leaky. Alternatively, protection by cytochrome *bd* may be due to its lack of proton pump functionality. Cytochrome *bd* in *E. coli* has been found electrogenic, but displays a low H^+^/e^-^ ratio^49^,^50^. In this way cytochrome *bd* may alleviate membrane hyperpolarization.

Inactivation of cytochrome *bd* converts the bacteriostatic activity of clofazimine and bedaquiline against *M. smegmatis* into strong bactericidal activity. This finding may be of pharmaceutical and clinical relevance as the bacteriostatic activity of bedaquiline is not restricted to *M. smegmatis*, but also found for pathogenic non-tuberculous mycobacterial strains, such as the *M. avium* complex^51^. These pathogenic strains typically show only low susceptibility towards current antibacterial chemotherapy^52^. Inactivation of cytochrome *bd* may assist in improving treatment options for infections caused by these recalcitrant bacteria. It would be important to assess if cytochrome *bd* deletion mutants in these pathogenic bacteria display increased sensitivity to (ROS-producing) antibacterials as well.

Inhibition of mycobacterial cytochrome *bd* by aurachin D serves as proof-of-concept for small-molecule inhibition of this important new drug target. Improved aurachin derivatives with better ability to penetrate the mycobacterial cell envelope may constitute a new class of anti-tubercular drugs. Cytochrome *bd* is of particular interest as potential drug target, as it is only found in prokaryotes. The absence of a human homologue may facilitate selective targeting. However, whole-cell screening on bacteria under aerobic, replicating conditions, which typically are applied for high-throughput discovery procedures^53^, may not allow for detection of cytochrome *bd* inhibition. Screening for bacteria under stressed conditions, e.g. in the presence of hydrogen peroxide or bedaquiline, may be applied as an alternative. Additionally, target- or pathway-based screenings, e.g. based on the inverted membrane vesicle system described in this report, against chemical libraries might lead to the discovery of potent cytochrome *bd* inhibitors.

## Materials & Methods

### Chemicals

Bedaquiline was obtained from Janssen, Pharmaceutical Companies of Johnson & Johnson. Aurachin D was a kind gift from Dr. Jennifer Herrmann (Helmholtz Centre for Infection Research and Pharmaceutical Biotechnology, Saarbrücken). All other chemicals were bought from Sigma unless indicated otherwise.

### Bacterial strains and growth conditions

*M. smegmatis* mc^2^ 155 was kindly provided by B.J. Appelmelk, Department of Molecular Cell Biology & Immunology, VU University Medical Center Amsterdam, The Netherlands. *M. smegmatis* mc^2^155 mutants Δ*qcrCAB* :: hyg and Δ*cydA* :: kan were kindly provided by Dr. B. Kana, MRC/NHLS/WITS Molecular Mycobacteriology Research Unit, National Health Laboratory Service, Johannesburg, South Africa. Replicating bacterial cultures were grown in Middlebrook 7H9 broth (Difco) supplied with 0.05% Tween-80 and 10% Middlebrook albumin dextrose catalase enrichment (BBL) at 37 °C with shaking. If applicable, 50 μg/mL kanamycin or 50 μg/mL hygromycin was added to the medium to select for mutant strains.

### Growth curves

Growth curves for wild-type and mutant *M. smegmatis* were determined using a 96-well plate system. Bacteria were diluted to an optical density at 600 nm of 0.01 and optical density was determined at 20 minute intervals for 60 hours. The optical density was measured with a UV-VIS spectrophotometer (Varian Cary50).

### Preparation of inverted membrane vesicles

Inverted membrane vesicles (IMVs) of the bacterial strains were prepared as described previously^54^. Briefly, *M. smegmatis* was grown for three days in a pre-culture to late-exponential phase. Cells were sedimented by centrifugation at 6000 x *g* for 20 minutes. The pellet was washed with phosphate buffered saline (PBS, pH 7.4) and centrifuged at 6000 x *g* for 20 min. Each 5 g of cells (wet weight) was re-suspended in 10 mL of ice-cold lysis buffer (10 mM HEPES, 5 mM MgCl_2_ and 10% glycerol at pH 7.5) including protease inhibitors (complete, EDTA-free; protease inhibitor cocktail tablets from Roche). Lysozyme (1.2 mg/mL), deoxyribonuclease I (1500 U, Invitrogen) and MgCl_2_ (12 mM) were added and cells were incubated with shaking for one hour at 37 °C. The lysates were passed three times through a One Shot Cell Disruptor (Thermo Electron, 40 K) at 0.83 kb to break up the cells. Unbroken cells were removed by three centrifugation steps (6000 x *g* for 20 min at 4 °C). The membranes were pelleted by ultracentrifugation at 222,000 x *g* for one hour at 4 °C. The pellet was re-suspended in lysis buffer and snap-frozen until use. The protein concentration was measured using the BCA Protein Assay kit (Pierce) as described by the manufacturer.

### Oxygen respiration assays

Oxygen respiration and the effect of inhibitors on oxygen respiration were measured by polarography using a Clark-type electrode. The electrode was fully aerated (212 μM O_2_ at 37 °C) and calibrated with sodium hydrosulfite. The inverted membrane vesicles were pre-incubated for three minutes with the inhibitors in a pre-warmed (37 °C) buffer containing 50 mM MES and 2 mM MgCl_2_ (pH 6.5). NADH was added as electron donor to a final concentration of 250 μM and oxygen respiration was measured for 90 seconds. Potassium cyanide was used as a control for inhibition. Two independent experiments were performed and average values plus standard errors were calculated.

**Cellular ATP levels** were determined using the luciferase bioluminescence method described previously^55^. Briefly, 1.0-mL samples taken from *M. smegmatis* cultures grown as described above were centrifuged at 8000 * g for 10 min. The pellets were re-suspended in 50 μl water and a 10-fold volume of boiling 100 mM TRIS-HCl, 4 mM EDTA (pH 7.75) was added. After incubation at 100 °C for 2 min the samples were centrifuged (1000 * g, 60 s) and the supernatants transferred to fresh tubes. 100 μl luciferase reagent (ATP Bioluminescence assay, Roche) was added to 100 μl sample and luminescence was measured with a Luminometer (LKB).

### Hydrogen peroxide and antibiotic sensitivity assays

Bacterial strains were grown to an optical density at 600 nm of 0.5. For hydrogen peroxide sensitivity assays, hydrogen peroxide (30% (w/v) stock) was added to an Eppendorf tube containing 0.49 mL of bacterial suspension to a final concentration of 20 mM. After the indicated time of incubation at 37 °C with shaking, 15 μl of catalase (10 mg/mL) was added to degrade hydrogen peroxide and thereby stop the reaction. For antibiotic sensitivity assays, 10 mL of bacterial cultures were incubated with the antibiotic for three (clofazimine and chlorpromazine) or four days (bedaquiline) at 37 °C with shaking. All samples were diluted in PBS and 0.1 mL was plated on 7H10 agar plates, containing oleic acid (0.05 g/l) and 10% Middlebrook albumin dextrose catalase enrichment (BBL). Cell viability was measured by counting colony-forming units per mL (CFU/mL) after 72 h (wild-type and Δ*cydA* :: kan strain) or 96 h (Δ*qcrCAB* :: hyg strain) incubation at 37 °C. The limit of detection was 100 CFU/mL. Survival was determined as percentage of surviving cells compared to untreated cells at day 0.

### ROS detection assays

For detection of reactive oxygen species the Amplex Red^®^ Hydrogen Peroxide/ Peroxidase Assay kit (Invitrogen) was used as described by the manufacturer with minor modifications. To measure ROS production in inverted membrane vesicles, 1 mL samples of 0.05 M sodium phosphate, pH 7.4 containing 20 μg *M. smegmatis* inverted membrane vesicles, 0.2 mM NADH, 50 μM Amplex Red^®^, 2 U horseradish peroxidase (HRP), 80 U superoxide dismutase (SOD) and the antibiotic diluted in DMSO in 1x reaction buffer (0.05 M sodium phosphate, pH 7.4) were prepared. Superoxide dismutase was added to allow for detection of superoxide. ROS production was determined by measuring absorbance at 563 nm for 30 minutes with a UV-VIS spectrophotometer (Varian Cary50).

## Additional Information

**How to cite this article**: Lu, P. *et al*. The cytochrome *bd*-type quinol oxidase is important for survival of *Mycobacterium smegmatis* under peroxide and antibiotic-induced stress. *Sci. Rep*. **5**, 10333; doi: 10.1038/srep10333 (2015).

## Supplementary Material

Supplementary Information
