# Supplementary material for: The cytochrome bd-type quinol oxidase is important for survival of Mycobacterium smegmatis under peroxide and antibiotic-induced stress
Source: Sci Rep. 2015 May 27;5:10333. doi: 10.1038/srep10333 (PMC4450806; doi:10.1038/srep10333)
Supplement: Supplementary Information [file srep10333-s1.doc]

**The cytochrome *bd*-type quinol oxidase is important for survival of *Mycobacterium smegmatis* under peroxide**

**and antibiotic-induced stress**

Ping Lu^1^, Marieke H. Heineke^1^, Anil Koul^2^, Koen Andries^2^, Gregory M. Cook^3^, Holger Lill^1^, Rob van Spanning^1^ & Dirk Bald^1^

^1^Department of Molecular Cell Biology, Amsterdam Institute for Molecules, Medicines and Systems, Faculty of Earth- and Life Sciences, VU University Amsterdam, De Boelelaan 1085, 1081 HV Amsterdam, The Netherlands,

^2^Infectious diseases and vaccines therapeutic area, Janssen Research & Development, Johnson & Johnson Pharmaceuticals, Turnhoutseweg 30, 2340-Beerse, Belgium,

^3^Department of Microbiology and Immunology, Otago School of Medical Sciences, University of Otago, Dunedin 9054, New Zealand.

A


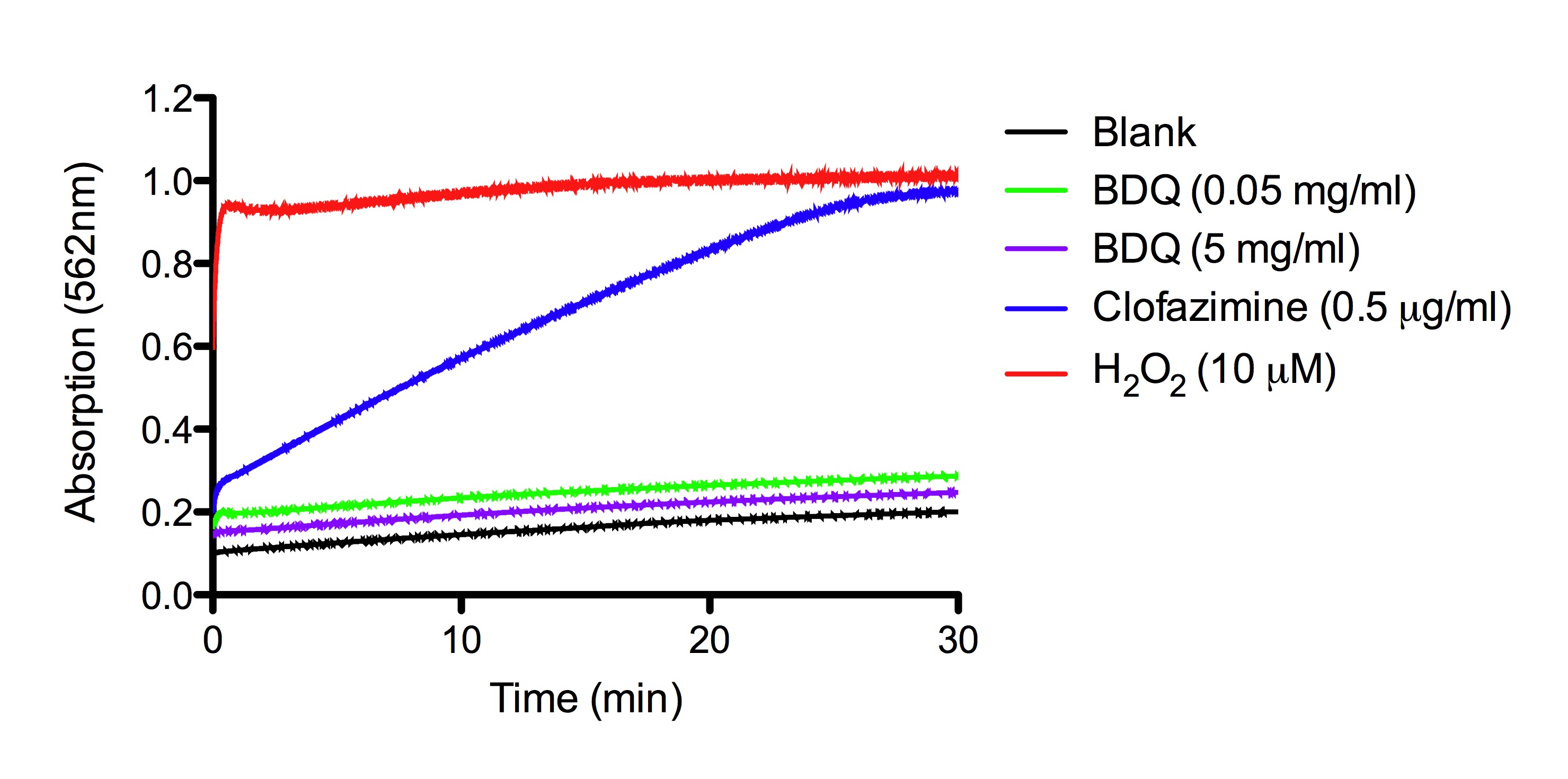


B


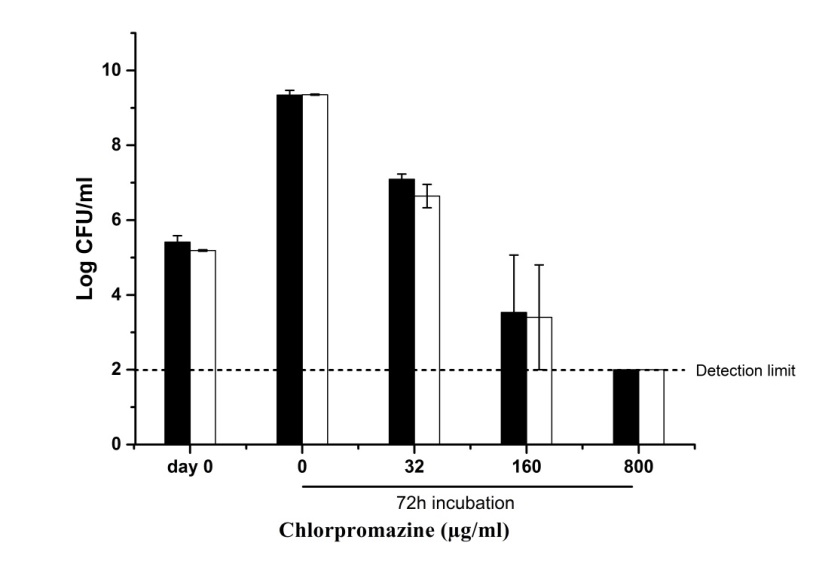


**Supplementary Figure 1: Mechanism of protection by cytochrome *bd*. (A)** ROS production by clofazimine and BDQ. ROS production was measured for 30 min after adding the indicated drugs to inverted membrane vesicles of wild-type *M. smegmatis*. The production of resofurin, a product of the 1:1 reaction of H_2_O_2_ with Amplex Red®, was measured at 562 nm. Representative results from two independent experiments are shown. **(B)** Effect of chlorpromazine on wild type and mutant *M. smegmatis* strains. *M. smegmatis* was treated with indicated amounts of chlorpromazine (CPZ) for 72 hours and CFU/ml were counted on agar plates after three days of incubation at 37°C. Black bars: WT; white bars: Δ*cydA*::kan. Results represent means of at least two independent experiments with standard error of the mean (SEM).
